# Features of Extrinsic Plantar Muscles in Patients with Plantar Fasciitis by Ultrasound Imaging: A Retrospective Case Control Research

**DOI:** 10.3390/diagnostics12040897

**Published:** 2022-04-04

**Authors:** Lorena Canosa-Carro, Daniel López-López, Fernando García-Sanz, Raquel Díaz-Meco-Conde, Paula García-Bermejo, Blanca de-la-Cruz-Torres, Jolanta Marszalek, Carlos Romero-Morales

**Affiliations:** 1 Faculty of Sport Sciences, Universidad Europea de Madrid, Villaviciosa de Odón, 28670 Madrid, Spain; lorena.canosa.c@gmail.com (L.C.-C.); raquel.diazmeco@universidadeuropea.es (R.D.-M.-C.); paulagarber91@gmail.com (P.G.-B.); carlos.romero@universidadeuropea.es (C.R.-M.); 2 Research, Health and Podiatry Group, Department of Health Sciences, Faculty of Nursing and Podiatry, Industrial Campus of Ferrol, Universidade da Coruña, 15403 Ferrol, Spain; 3 Department of Physiotherapy, Clínica CEMTRO, 28035 Madrid, Spain; fernando.garcia@clinicacemtro.com; 4 Department of Physiotherapy, University of Seville, Avicena Street, 41009 Seville, Spain; bcruz@us.es; 5 Faculty of Rehabilitation, Jozef Pilsudski University of Physical Education, 00-809 Warsaw, Poland; jolanta.marszalek@awf.edu.pl

**Keywords:** ultrasonography, plantar fasciitis, diagnostics, imaging

## Abstract

The present study aimed to compare by ultrasound imaging (USI) the tibial posterior (TP), medial gastrocnemius (MG) and soleus muscle in patients with and without plantar fasciitis (PF). A sample of 42 individuals was recruited and divided into two groups: PF and a healthy group. The thickness, cross-sectional area (CSA), echointensity and echovariation were assessed in both groups by USI. TP, soleus and MG variables did not report differences (*p* > 0.05) for thickness and CSA. For the echotexture parameters significant differences were found for MG echointensity (*p* = 0.002), MG echovariation (*p* = 0.002) and soleus echointensity (*p* = 0.012). Non-significant differences (*p* > 0.05) were reported for soleus echovariation, TP echointensity and TP echovariation variables. The thickness and CSA of the TP, GM and soleus muscle did not show significant differences between individuals with and without PF measured by USI. Muscle quality assessment reported an increase of the MG echointensity and echovariation, as well as a decrease of echointensity of the soleus muscle in the PF group with respect to the healthy group. Therefore, the evaluation of the structure and muscle quality of the extrinsic foot muscles may be beneficial for the diagnosis and monitoring the physical therapy interventions.

## 1. Introduction

Objective information about the muscle and soft tissues morphology by ultrasound imaging (USI) was considered very useful in diagnostics and rehabilitation programs follow-up [1]. In this line, USI was employed to assess musculoskeletal disorders, exercise effects in patients with neurological conditions, to provide an imaging guidance of minimally-invasive techniques in physical therapy (e.g., dry needling or percutaneous neuromodulation), and also for the assessing of the muscle fatty infiltration [2,3]. Measurements of thickness, cross-sectional area (CSA), angle of pennation, fascicle length, and muscle quality parameters (e.g., echotexture) were considered as very important information within the musculoskeletal broad context [2].

Over recent years, the use of USI has been increasing by physical therapists as a diagnostic and research tool, and also to guide invasive procedures [4]. Several authors reported the benefits to quantify the muscle architecture and textures of the lower limb with USI; for example, Taniguchi et al. reported a reduced thickness of the vastus medialis in patients with knee osteoarthritis [5]. Calvo–Lobo showed a reduced CSA of the peroneus longus in subjects who suffer ankle sprains [6]. USI evaluations of the abductor hallucis and flexor digitorum brevis reported an increase of the thickness in patients with Achilles tendinopathy (AT) [7]. Romero et al. also found a reduced CSA of the extensor digitorum longus (EDL), tibialis anterior (TA) and peroneus (PER) muscles in individuals with AT [8]. USI analysis using quantitative ultrasound descriptors have been employed to asses texture parameters based on the gray-scale pixel distribution from a selected region of interest (ROI) [9,10]. Echointensity (EI) was described as the mean value of the pixel intensity [11]. This parameter has been linked to muscle quality, reporting cellular changes in musculoskeletal conditions [11]. Echovariation (EV) has also been described as an statistical parameter to evaluate the pixel distribution taking in consideration the standard deviation (SD) of the histogram values from the EI [3]. Furthermore, several studies showed the benefits of the musculoskeletal system assessment through echotextural parameters [3,12].

Regarding the foot and ankle musculoskeletal structures and conditions, plantar fasciitis (PF) is one of the most prevalent causes of heel pain, affecting an estimated 10% of the general population [13]. PF was also considered a degenerative process associated with pain, lack of functionality and stiffness in the plantar fascia. Patients diagnosed with PF reported the main symptoms usually in two specific locations: at the calcaneal insertion and in the mid-foot area [14]. As noted above, PF is considered an overuse injury, being repetitive trauma or load mismanagements the main etiology factors for its development [15]. In addition, episodes might be accompanied with or without inflammation [16]. Cotchett et al. argued that individuals with PF feel pain within the first steps in the morning or after inactive periods [17]. Several intrinsic risk factors are known: high body mass index (BMI), biomechanics alterations such as a restricted ankle dorsiflexion, pes planus or pes cavus, excessive foot pronation and weakness of the plantar foot muscles [18]. Some extrinsic risk factors to PF are inadequate footwear, high physical activity demands, poor-quality surfaces and walking barefoot [18].

Angin et al. explained the importance of the extrinsic foot muscles in the foot and ankle biomechanics, TA, EDL and PER muscles acts in a coordinated manner with the intrinsic foot muscles in order to provide stability and control of the foot and ankle joint [19]. For example, the PER muscles have an important role on the stability of the subtalar joint, acting as a plantar flexor to provide the eversion required to maintain the opposite foot inversion activity [20]. In the same line, Balen and Helms argued that an injury of the tibial posterior (TP) muscle and tendon could produce a failure on the loading foot system with a decrease of the medial arch. Individuals with TP tendon insufficiency might develop disturbances on the spring ligaments and plantar fascia [21]. The posterior complex formed by the gastrocnemius (both medial and lateral) and soleus muscle, work in a coordinated manner with the Achilles tendon as a musculotendinous unit which provides acceleration and deceleration forces to the ankle joint during the gait cycle. Pascual-Huerta reported that an increase of the gastrocnemius muscle tightness was related with an increase of the Achilles tendon tension which could produce an increase of the tension on the plantar fascia during weight-bearing activities [22]. In this line, the interaction of the gastrocnemius and soleus muscle with the plantar fascia was evidenced from a mechanical view on the sagittal plane [22] As stated above, both structures also play an important role on the ankle and foot stabilization, weight-bearing and balance moments [22]. Thus, alterations on the medial and posterior extrinsic foot muscles architecture could produce ankle and foot musculoskeletal disturbances, such as PF. The aim of the present study was to compare by USI the TP, medial gastrocnemius (MG) and soleus muscle in patients with and without PF. Authors hypothesized that in presence of PF, the muscle architecture of the posterior extrinsic foot muscles is altered.

## 2. Materials and Methods

### 2.1. Design

A case-control research study was developed according to the Strengthening the Reporting Observational studies in Epidemiology (STROBE) guidelines [23]. This study was approved by the ethics committee of Universidad Europea code: CIPI/20/166. All the participants signed an informed consent form before the study participation. In addition, the Helsinki Declaration was respected from the start to until the end of the study.

### 2.2. Participants

The research sample consisted in 42 participants recruited from August to September 2021. The sample was divided in two groups: A group formed by 21 participants diagnosed with PF by a specialized medical doctor with more than 15 years of experience, based on the following inclusion criteria: heel pain of at least 1 month, pain with tenderness on palpation located in the middle of the plantar fascia or at the medial calcaneal tubercle, pain during the first septs in the morning or after non-weight bearing activities [24,25]. Exclusion criteria included subjects with systemic diseases, fractures, low back pain, previous lower limb surgeries or infection, length leg differences greater 1 cm and other musculoskeletal condition in the last year (e.g., ankle sprain, Achilles tendinopathy) [26].

The sample size calculation was performed by the G*Power software to compare the difference between the PF and healthy group employing the TP CSA variable of a pilot study (*n* = 8) that was divided into two groups (mean ± SD): 4 participants with PF (1.42 ± 0.35) and 4 participants for the healthy group (1.75 ± 0.48). For the sample size calculation, a power of 0.80, an α error of 0.05 and an effect size of 0.78 with a one-tailed hypothesis were employed. In conclusion, a sample of 42 was calculated.

### 2.3. Ultrasonography Examination

**Figure 1:**
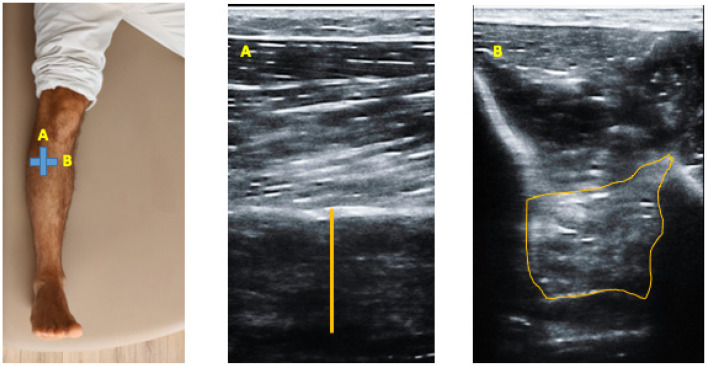
Ultrasound imaging thickness and CSA of the TP muscle. (**A**) Upper third of the imaginary line in a longitudinal view (thickness) and (**B**) Transversal view (CSA) to identify the TP.

**Figure 2:**
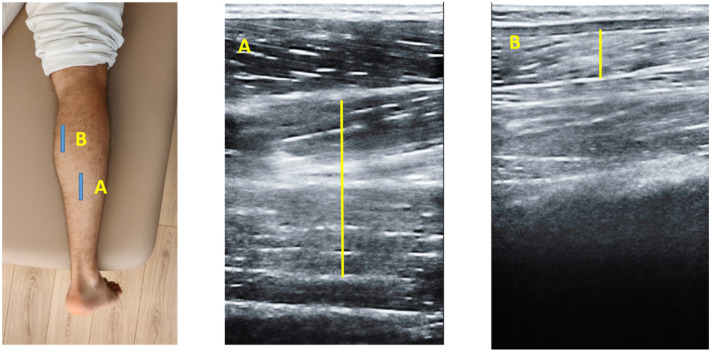
Ultrasound imaging of the MG and soleus. (**A**) Longitudinal view into the soleus and gastrocnemius intersection and (**B**) View MG longitudinal axis of the muscle where located the transducer along-side.

Ultrasonography assessment was developed by a high-quality ultrasound system Mindray DC-60 with a 6 to 14 MHz linear transducer (L14-6NE) in B-Mode. All the USI evaluations were performed by the same clinician with more than 3 years of experience in ultrasound imaging (L.C.C). To the TP evaluations, according to Wayne Johnson et al. participants were lying in a supine position [27]. To assess the TP thickness and the CSA an imaginary line from the lateral knee joint and the lateral malleolus was defined and place the transducer in the upper third of the imaginary line in a longitudinal view (thickness) (Figure 1A) and in a transversal view (CSA) (Figure 1B) to identify the TP [27]. For the MG and soleus muscle measurements, participants were placed in prone position and the transducer was located in longitudinal view into the soleus and gastrocnemius intersection (Figure 2A) and for the MG the transducer was located alongside the longitudinal axis of the muscle (Figure 2B) [28].

### 2.4. Image Analysis

The imaging study was developed offline by the ImageJ software (Bethesda, MD, USA). The mean of three repeated values was recorded for each measurement and analyzed for the thickness and CSA variables.

According to previous research, the EI and EV variables were extracted from a region of interest (ROI) of 64 × 64 with an 8-bit gray-scale to show the pixel distribution histogram. ROI was defined as the muscle area without bone and fascia to reach the best reflection [29]. Echotexture parameters were captured from the pixel distribution histogram and as stated above EI was described as the mean value of the gray-scale pixel distribution and EV as the relation between the mean and SD of the pixel distribution [EV = (SD/mean) × 100].

### 2.5. Data Analysis

Statistical analysis was developed with the SPSS software (v.21, IBM, Armonk, NY, USA). Shapiro-Wilk test was employed to check the normality distribution. Descriptive analysis of the total sample and divided in two groups were performed. The mean, standard deviation (SD) and Student’s *t*-test for independent groups was performed for parametric data and median, interquartile range (IR) and Mann-Withney *U* test were employed for non-parametric data. In addition, Levene-s test was employed to assess the equality of variances. An α error of 0.05 (95% CI) and a power of 80% (β error of 0.2) were carried out for all statistical tests.

## 3. Results

Regarding Table 1, sociodemographic data showed differences in height (*p* = 0.001) and in BMI (*p* = 0.017) between groups. According to the Table 2, TP, soleus and MG variables did not report differences (*p* > 0.05) for thickness and CSA. For the echotexture parameters significant differences were found for MG EI (*p* = 0.002), MG EV (*p* = 0.002) and soleus EI (*p* = 0.012). In addition, non-significant differences (*p* > 0.05) were reported for soleus EV, TP EI and TP EV variables.

## 4. Discussion

The goal of the present study was to investigate the differences between groups (PF and healthy) by USI of the extrinsic foot muscle structure (thickness and CSA) and muscle quality (EI and EV). Results reported differences in echotexture between groups according to the ultrasonography pattern. Regarding the structural parameters, no differences were described in thickness and CSA for each variable for TP, MG and soleus muscle. These variables were of interest due to the relationship between extrinsic foot muscles and foot musculoskeletal disorders such as PF. Several authors have remarked the importance of the pressure load transmission system on account of an 83% of that load being transmitted through the tibia and ankle, and foot structures while a 17% is transmitted to the fibula [30,31,32]. Thus, the muscles directly related to this area, such TP, GM and soleus muscle could be susceptible to ultrasonography patterns changes in patients who suffer PF. Despite the results of the present study were not able to found differences between groups in structure ultrasound parameters, authors think that could be convenient the assessment of the extrinsic foot muscles due to the compensatory forces in ankle and foot biomechanics (e.g., excessive pronation, muscle weakness) in patients with PF and surrounding ankle and foot tissues structures [18]. For example, tibial rotations were directly related with Achilles tendon disturbances [33]. Maffulli et al. reported that an excessive pronation could be considered a risk factor for the development of alterations in ankle and foot biomechanics, such Achilles tendon disturbances, which is intimately related with PF [34] In this line, a decrease on the plantar fascia thickness in individuals with Achilles tendinopathy were also reported [35].

Considering the muscle quality analysis to evaluate tissue homogeneity our results showed an increase of MG EI which could be explained by the muscle adaptations produced in the extrinsic foot muscles for the pathology, such as fat an collagen infiltration [36]. Supporting this hypothesis, several authors suggested that an increase of EI parameter could be useful to assess the muscle quality in different situations, such as degenerative muscle disturbances [37,38]. Regarding the soleus EI parameter, the present study reported a decrease with respect to the healthy group. Several authors hypothesize that a minor EI could be related to a lack of structure and thus, a less homogeneity in the ROI due to the presence of a hypoechoic pattern [39,40,41].

Respect to the EV parameter, this study found an increase on the MG and no differences for the TP and soleus muscles. Contrarily to our results, Martinez-Payá et al. reported a decrease in the EV of the biceps brachialis, quadriceps femoris and tibialis anterior in individuals diagnosed with amyotrophic lateral sclerosis [42]. In this context, authors hypothesized with the possibility of providing a new muscle biomarker with ultrasonography to clinicians and researchers.

### 4.1. Limitations and Future Lines

There are several limitations to this study. First, BMI variable reported differences between groups which may had repercussion on the ultrasonography muscle textures. Second, USI assessment was not made by a blinded researcher; moreover ultrasonography quality is operator dependent and subjective to interpretative error. At last, the study was based in a small sample size, so the results of the present study should be interpreted with caution. More studies still needed to a better understanding of the extrinsic foot and ankle muscles in subjects diagnosed with PF. In addition, the implementation of the ultrasound M-mode could be useful to check the muscular activation and inhibition.

### 4.2. Clinical Applications

The most important clinical applications for the present study highlighted the benefits of the muscle quality parameters—EI and EV—as an objective complement for the USI examination of the musculoskeletal tissue. The use of ultrasonography approaches for the preventive and physical therapy management programs for musculoskeletal disorders, such as PF, should be considered by clinicians and researchers.

## 5. Conclusions

The thickness and CSA of the TP, GM and soleus muscle did not show significant differences between individuals with and without PF measured by USI. Muscle quality assessment reported an increase of the GM EI and EV as well as a decrease of EI of the soleus muscle in the PF group with respect to the healthy group. Therefore, GM muscle shows an altered muscle structure (e.g., fatty infiltration) and should be taken in consideration for the rehabilitation programs development. In addition, the evaluation of the structure and muscle quality of the extrinsic foot muscles may be beneficial for the diagnosis and monitoring the physical therapy interventions.

## Figures and Tables

**Table 1:** Sociodemographic data of the sample.

Data	Total Sample (*n* = 42)	Plantar Fasciitis (*n* = 21)	Controls (*n* = 21)	*p*-Value Cases vs. Controls
Age, y	39.23 ± 11.10	42.54 ± 11.57	36.95 ± 11.19	0.115
Weight, kg	77.80 ± 14.87	79.90 ± 27.00	75.13 ± 9.88	0.293
Height, m	1.72 ± 6.05	1.71 ± 6.3	1.76 ± 5.30	0.001
BMI, kg/m^2^	26.07 ± 4.80	27.66 ± 5.27	24.27 ± 3.44	0.017

Abbreviations: BMI, body mass index.

**Table 2:** Ultrasound imaging measurements of the intrinsic muscles thickness, CSA, EI and EV.

Measurement	Plantar Fasciitis (*n* = 32)	Controls (*n* = 32)	*p*-Value
Distance (cm)			
TP CSA	1.43 ± 0.65	1.76 ± 0.56	0.375
TP Th	1.13 ± 0.30	1.17 ± 0.15	0.076
GM Th	1.57 ± 0.56	1.66 ± 0.34	0.679
Soleus Th	0.68 ± 0.21	0.71 ± 0.14	0.512
MG EI	104.14 ± 17.19	96.98 ± 14.48	0.015
MG EV	15.59 ± 6.42	19.42 ± 6.42	0.002
Soleus EI	85.52 ± 15.09	92.66 ± 15.49	0.012
Soleus EV	12.17 ± 4.66	12.49 ± 4.25	0.690
TP EI	88.04 ± 14.01	84.56 ± 13.74	0.173
TP EV	10.83 ± 3.23	12.81 ± 12.15	0.225

Abbreviations: cross-sectional area, CSA; echointensity, EI; echovariation, EV; mediales gastrocnemius, MG; Thickness, Th; Tibialis posterior, TP.

## Data Availability

Data available under formal request.
